# Correction: Teacher-student approach for lung tumor segmentation from mixed-supervised datasets

**DOI:** 10.1371/journal.pone.0298978

**Published:** 2024-02-13

**Authors:** Vemund Fredriksen, Svein Ole M. Sevle, André Pedersen, Thomas Langø, Gabriel Kiss, Frank Lindseth

There are errors in the Funding section. The correct Funding statement is: This work was supported by The Norwegian National Advisory Unit for Ultrasound and Image-Guided therapy at St. Olavs hospital, The Liaison Committee for Education, Research and Innovation in Central Norway (No. 2018/42794), The Cancer Foundation, St. Olavs hospital, Trondheim University Hospital (No. 13/2021), SINTEF, and the Norwegian Financial Mechanism 2014–2021 under the project RO- NO2019-0138, 19/2020 “Improving Cancer Diagnostics in Flexible Endoscopy using Artificial Intelligence and Medical Robotics” IDEAR, Contract No. 19/2020.
